# Vapor Pressure and Enthalpy of Vaporization of Guanidinium Methanesulfonate as a Phase Change Material for Thermal Energy Storage

**DOI:** 10.3390/ma17112582

**Published:** 2024-05-27

**Authors:** Wenrong Bi, Shijie Liu, Xing Rong, Guangjun Ma, Jiangshui Luo

**Affiliations:** Laboratory of Electrolytes and Phase Change Materials, College of Materials Science and Engineering, Sichuan University, Chengdu 610065, China

**Keywords:** organic salt, ionic liquid, guanidinium methanesulfonate, recrystallization, thermal property, vapor pressure, enthalpy of vaporization, phase change material, thermal energy storage

## Abstract

This paper reports the vapor pressure and enthalpy of vaporization for a promising phase change material (PCM) guanidinium methanesulfonate ([Gdm][OMs]), which is a typical guanidinium organomonosulfonate that displays a lamellar crystalline architecture. [Gdm][OMs] was purified by recrystallization. The elemental analysis and infrared spectrum of [Gdm][OMs] confirmed the purity and composition. Differential scanning calorimetry (DSC) also confirmed its high purity and showed a sharp and symmetrical endothermic melting peak with a melting point (*T*_m_) of 207.6 °C and a specific latent heat of fusion of 183.0 J g^−1^. Thermogravimetric analysis (TGA) reveals its thermal stability over a wide temperature range, and yet three thermal events at higher temperatures of 351 °C, 447 °C, and 649 °C were associated with vaporization or decomposition. The vapor pressure was measured using the isothermogravimetric method from 220 °C to 300 °C. The Antoine equation was used to describe the temperature dependence of its vapor pressure, and the substance-dependent Antoine constants were obtained by non-linear regression. The enthalpy of vaporization (Δ_vap_*H*) was derived from the linear regression of the slopes associated with the linear temperature dependence of the rate of weight loss per unit area of vaporization. Hence, the temperature dependence of vapor pressures ln *P*_vap_ (Pa) = 10.99 − 344.58/(*T* (K) − 493.64) over the temperature range from 493.15 K to 573.15 K and the enthalpy of vaporization Δ_vap_*H* = 157.10 ± 20.10 kJ mol^−1^ at the arithmetic mean temperature of 240 °C were obtained from isothermogravimetric measurements using the Antoine equation and the Clausius–Clapeyron equation, respectively. The flammability test indicates that [Gdm][OMs] is non-flammable. Hence, [Gdm][OMs] enjoys very low volatility, high enthalpy of vaporization, and non-flammability in addition to its known advantages. This work thus offers data support, methodologies, and insights for the application of [Gdm][OMs] and other organic salts as PCMs in thermal energy storage and beyond.

## 1. Introduction

**Figure 1:**
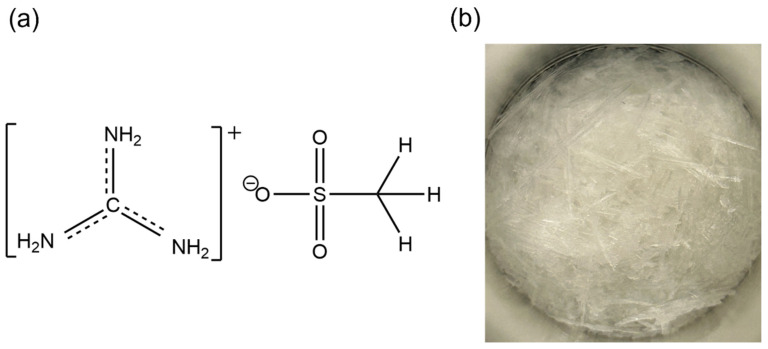
The chemical structure of [Gdm][OMs] (**a**) and the photograph of its crystals (**b**).

Thermal energy storage technologies employing phase change materials (PCMs) offer a promising solution for the intermittency of solar energy and industrial waste heat recovery and utilization [1,2,3,4,5]. PCMs can be divided simply into two categories: inorganic PCMs and organic PCMs [5]. Table 1 lists some PCMs with melting points in the intermediate temperature range between 100 °C and 230 °C. Among them, inorganic salt hydrates (e.g., MgCl_2_·6H_2_O) have poor cycling stability due to water separation and high supercooling degree [6]. Metals such as indium and selenium are unsuitable for use as large-scale PCMs due to their low latent heat of fusion (Δ_fus_*H*) [7]. Organic PCMs such as erythritol and D-dulcitol have high values of Δ_fus_*H*, but their significant supercooling is not conducive to heat release [8,9,10,11]. As a new kind of PCM, ionic liquids (or organic salts) have unique physicochemical properties, including non-flammability, low volatility, excellent thermal stability, tunable melting points, and high heat storage density, offering potential applications in thermal energy storage [12,13]. Currently, protic ionic liquids derived from pyridines [14], guanidines [15,16], pyrazoles [13], and imidazoles [12,17] have been explored as potential PCMs. Among them, guanidinium methanesulfonate ([Gdm][OMs], i.e., guanidinium mesylate, Figure 1) exhibits the highest Δ_fus_*H* to date (*T*_m_ = 208 °C, Δ_fus_*H* = 190 J g^−1^, Table 1) with total volumetric energy storage measured as 622 MJ m^−3^ (173 kWh m^−3^) and an excellent cyclic stability after 420 cycles between 150 °C and 215 °C, making it the state-of-the-art PCM based on protic organic salts [15,16]. Overall, [Gdm][OMs] has been identified as a very promising PCM for inexpensive renewable energy storage applications at intermediate temperatures [7,15,16,18]. Moreover, [Gdm][OMs] is a representative guanidinium organomonosulfonate (GMS), which displays a series of lamellar crystalline architectures and a two-dimensional hydrogen-bonding network of complementary guanidinium ions (G) and sulfonate moieties (S), the so-called GS sheet [19]. Previous studies on [Gdm][OMs] as a PCM in terms of thermal energy storage include melting temperature, latent heat of fusion, heat capacity, thermal conductivity, volume change, advanced thermal stability, long-term cycling, and economic analysis [15,16]. However, so far, there has been no report on the vapor pressure and vaporization enthalpy of [Gdm][OMs] to the best of our knowledge.

The determination of the temperature-dependent vapor pressure of ionic liquids (ILs) is useful for their industrial application. A higher vapor pressure of the storage medium can lead to thicker walls and the potential loss of PCMs, increasing cost [5], and safety concerns for thermal energy storage containers. Hence, vapor pressure (*P*_vap_) and enthalpy of vaporization (Δ_vap_*H*) are not only important basic thermal properties but also key parameters for designing thermal energy storage systems, which are critical for the long-term safety of PCMs and thermal energy storage systems. In addition, Δ_vap_*H* reflects molecular interactions in the liquid state and can serve as a foundation for calibrating and validating force fields in molecular dynamics simulations and as an anchoring parameter in *P*−*V*−*T* equations for neat ILs [20,21]. For example, Liu et al. [21] calculated the values of Δ_vap_*H* of tetrabutylammonium bis(trifluoromethanesulfonyl)imide ([N_4444_][NTf_2_]) and investigated its cohesive energy, enthalpy, and entropy. The Gibbs free energy of the transition from condensed state to gas was successfully obtained using Born–Fajans–Haber cycles.

In this work, the protic organic salt [Gdm][OMs] was prepared and purified via recrystallization (Figure 1). Its composition and purity were verified by elemental analysis and Fourier transform infrared spectroscopy (FT-IR). The melting temperature (*T*_m_), latent heat of fusion (Δ_fus_*H*), and thermal decomposition temperature (*T*_d_) were studied by differential scanning calorimetry (DSC) and thermogravimetric analysis (TGA). *P*_vap_ and Δ_vap_*H* of [Gdm][OMs] were determined using the isothermogravimetric method. The functional relationship between vapor pressure and temperature was fitted using the Antoine equation. [Gdm][OMs] exhibits very low vapor pressure, high enthalpy of vaporization, and non-flammability in addition to its reported merits like high thermal and cyclic stability, offering data support for its application as a PCM in thermal energy storage.

## 2. Experimental

### 2.1. Materials

The materials used in this study were as follows: guanidinium carbonate ((CH_5_N_3_)_2_·H_2_CO_3_, 99%, Acros Organics, Geel, Belgium), methanesulfonic acid (CH_3_SO_3_H, 99%, Sigma-Aldrich, Steinheim, Germany), ethanol (200 proof, anhydrous, ≥99.5%, Sigma-Aldrich, St. Louis, MO, USA), and glycerol (HOCH_2_CHOHCH_2_OH, ≥98%, Beijing Solarbio Science & Technology Co., Ltd., Beijing, China). They were all utilized without further purification.

### 2.2. Synthesis of the Organic Salt

The synthesis of guanidinium methanesulfonate ([Gdm][OMs], i.e., guanidinium mesylate) involves the slow addition of 22.61 mL of methanesulfonic acid to 15.69 g of guanidinium carbonate in a round bottom flask at room temperature. It should be noted that the adopted ratio of these two raw materials (CH_3_SO_3_H:(CH_5_N_3_)_2_·H_2_CO_3_) slightly exceeds the stoichiometric ratio of 2:1, ensuring excess methanesulfonic acid and thus the complete reaction of the guanidinium carbonate. Then, the resulting wet slurry was heated to 210 °C in an oil bath to form a homogenous melt, followed by stirring at 210 °C via a magnetic stirrer for 5 h to promote the complete formation of the expected salt. The reaction was accompanied by the production of large quantities of gas and water. Most of the water was evaporated off during the reaction while the excess methanesulfonic acid was removed under vacuum at 250 °C.

### 2.3. Recrystallization

The [Gdm][OMs] sample was purified by recrystallization before further use as follows: Dissolve the as-prepared [Gdm][OMs] in anhydrous ethanol in a sealed glass vial, and sonicate the solution in a water bath at 50 °C for 30 min until it is completely clear. The mass ratio of [Gdm][OMs] vs. anhydrous ethanol is about 1:15.0~15.4. Then, cool the solution and maintain it at 0 °C for 12 h. Afterward, the obtained crystals were filtered off and dried at 80 °C in an oven for 1 h.

### 2.4. Elemental Analysis

The composition of the [Gdm][OMs] sample was characterized using an elemental analyzer (UNICUBE, Elementar, Langenselbold, Germany) with a detection limit of less than 50 ppm using a thermally conductive detector.

### 2.5. Fourier Transform Infrared (FT-IR) Analysis

The FT-IR spectrum of the [Gdm][OMs] powders was recorded at room temperature in the range of 4000–400 cm^−1^ on a spectrometer (INVENIO-R, Bruker, Ettlingen, Germany) with a universal ATR accessory and was accumulated for 16 scans at a resolution of 4 cm^−1^.

### 2.6. Differential Scanning Calorimetry (DSC)

DSC measurements were conducted between 25 °C and 240 °C on a thermal analysis system (STAR^e^ DSC 3, Mettler-Toledo, Greifensee, Switzerland) at a heating and cooling rate of 5 °C min^−1^ to identify the phase transition temperatures and latent heat of fusion (Δ_fus_*H*). The values are reported using the second run of the DSC tests to eliminate possible thermal history. The melting point (*T*_m_) was fixed as the peak temperature and Δ_fus_*H* as the integrated area of the melting peak. Measurements were carried out using sealed aluminum pans (40 μL) under a nitrogen atmosphere (60 mL min^−1^) with a sample weight of 8.34 mg.

### 2.7. Thermogravimetric Analysis (TGA)

All thermogravimetric tests were carried out on the same instrument (TGA 2, Mettler Toledo, Columbus, OH, USA), including variable temperature thermogravimetric analysis and isothermogravimetric analysis (IGA). Concerning the ramped TGA measurement, the sample was tested from 30 °C to 850 °C under N_2_ (50 mL min^−1^) and at a heating rate of 5 °C min^−1^ with a sample weight of 11.07 mg using a covered Al_2_O_3_ pan. The decomposition temperature (*T*_d_) is determined by the onset temperature.

The IGA experiments of [Gdm][OMs] and glycerol were carried out on the same instrument using the same batch of uncovered Al_2_O_3_ crucible (cross-sectional area (a): 0.204 cm^2^) under the same nitrogen atmosphere (50 mL min^−1^). For [Gdm][OMs], it was conducted at 220 °C, 240 °C, 260 °C, 280 °C, and 300 °C successively. It was held for 30 min at each measurement temperature. The heating rate for each measurement temperature was 20 °C min^−1^. Similarly, the IGA experiments for glycerol were conducted at 100 °C, 120 °C, 140 °C, and 160 °C successively, and at each temperature it was held for 30 min. The masses of [Gdm][OMs] and glycerol were 10.40 mg and 42.36 mg, respectively.

### 2.8. Flammability Test

The flammability of [Gdm][OMs] was tested by direct ignition in a battery case for 5 s.

## 3. Theoretical Basis

### 3.1. Vapor Pressure

The vapor pressures of [Gdm][OMs] can be derived based on the Langmuir equation using the thermogravimetric data [22,23]:

(1) $$\left(-dm/dt\right)/S=P_{\mathrm{vap}}\alpha_{\mathrm{vap}}{\left(M_{w}/2\pi RT\right)}^{0.5}$$

where (−d*m*/d*t*)/*S* is the rate of mass loss of the sample per unit area of vaporization (g min^−1^ cm^−2^), *P*_vap_ is the vapor pressure (Pa), *α*_vap_ is the vaporization coefficient, *M_w_* is the molecular weight of the effusing vapor (g mol^−1^), *R* is the gas constant (8.314 J mol^−1^ K^−1^), and *T* is the absolute temperature (K). *α*_vap_ is considered to be dependent on the apparatus and experimental atmosphere rather than on the substance and temperature. Furthermore, it can be determined by using pure substances with reported precise vapor pressures [24,25]. Equation (1) is simplified as Equation (2):

(2) $$P_{\mathrm{vap}}=k_{\mathrm{vap}}v$$

where *k*_vap_ is a constant (Pa min cm^2^ g^−0.5^ mol^−0.5^ K^−0.5^), *v* is dependent on the material (g^0.5^ mol^0.5^ K^0.5^ min^−1^ cm^−2^), *k*_vap_ = (2πR)^0.5^/*α*_vap_, and *v* = [(−d*m*/d*t*)/*S*](*T*/*M*_w_)^0.5^.

Based on experimental data at several temperatures, vapor pressures in a given temperature range can be predicted by Antoine equation (Equation (3)) [26], which is an empirical equation that describes the *P*−*T* relationship of pure liquid substances and offers various applications in engineering:

(3) $$\ln P_{\mathrm{vap}}=A-\frac{B}{T+C}$$

where *A*, *B,* and *C* are the substance-dependent Antoine constants (also known as Antoine coefficients) and *P*_vap_ is the vapor pressure (Pa) at temperature *T* (K).

### 3.2. Enthalpy of Vaporization

Enthalpy of vaporization of substances can be calculated by Equation (4), which is a regression equation derived from the Clausius–Clapeyron equation and was successfully used by Luo et al. [27] to calculate the enthalpy of vaporization of imidazolium-based ionic liquids:

(4) $$\ln\left[(-dm/dt)T^{0.5}/S\right]=A-\frac{{∆}_{vap}H}{RT}$$

where (−d*m*/d*t*)/*S* is the rate of mass loss of the sample per unit area of vaporization (g min^−1^ cm^−2^), *T is* the absolute temperature (K), *R* is the gas constant (8.314 J mol^−1^ K^−1^), Δ_vap_*H* is the molar enthalpy of vaporization at the arithmetic mean temperature of all tested temperature points, and *A* is an empirical parameter. This method has recently been widely used to calculate Δ_vap_*H* of various ionic liquids [28,29,30].

## 4. Results and Discussions

### 4.1. Basic Characterizations

The as-synthesized [Gdm][OMs] was examined by elemental analysis. The experimental values of the elemental contents of C, H, N, S, and O were as follows (the calculated values based on the molecular formula are given in parentheses): C-16.06% (15.48%), H-5.98% (5.81%), N-27.73% (27.10%), S-20.07% (20.65%), and O-30.16% (30.96%). It can be seen that the experimental values are in line with the calculated values for all the five elements. The chemical composition of the tested sample can be inferred to be C_2_H_9_N_3_O_3_S, indicating the high purity of the as-prepared sample.

**Figure 2:**
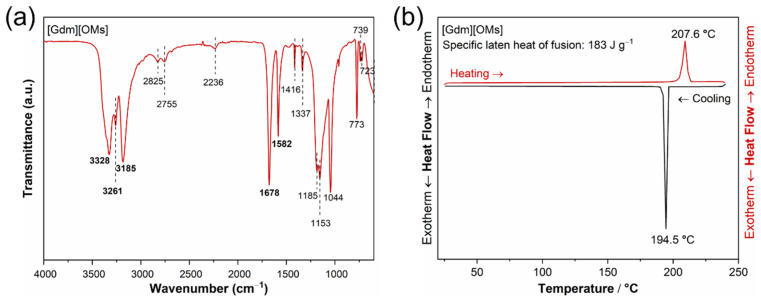
(**a**) FT-IR spectrum of [Gdm][OMs] in the region of 600–4000 cm^−1^. (**b**) DSC trace from the second heating/cooling cycle for [Gdm][OMs] (heating and cooling rate: 5 °C min^−1^).

The structure of the [Gdm][OMs] is also confirmed by FT-IR spectrum (Figure 2a). The appearance of new peaks that are different from the characteristic peaks of both guanidine carbonate and methanesulfonic acid, as well as the disappearance of some peaks, proved the formation of the proton transfer organic salt [31,32]. The as-prepared [Gdm][OMs] sample has a very low water content, indicated by the absence of the O−H stretching bands at 3400−3800 cm^−1^ and the H−O−H bending band at around 1650 cm^−1^ in the infrared spectrum [33,34,35]. Based on our previous work [32], the formation of CH_3_SO_3_^−^ anions in protic organic salts is confirmed by the bands at 1416, 1337, 1185, 1153, 1044, 773, 739, and 723 cm^−1^, while the existence of guanidinium cations in [Gdm][OMs] is evidenced by the peaks at 3328, 3261, 3185, 1678, and 1582 cm^−1^ (Figure 2a) [31,36]. The sharp absorption bands at around 1185 and 1153 cm^−1^ are assigned to the SO^3−^ asymmetric stretching vibration, while the peak at 1044 cm^−1^ relates to the SO^3−^ symmetric stretching vibration [37]. This indicates a transformation of the −SO_2_OH group into the −SO^3−^ anionic group, implying the occurrence of a proton transfer process from the acidic site to the basic site. This spectral feature is typical of CH_3_SO_3_^−^ anions strongly involved in hydrogen bonding [38]. The peaks at 3328, 3261, and 3185 cm^−1^ correspond to the asymmetric and symmetric NH_2_ stretching vibrations, respectively [36,39]. In addition, the peaks at 1678 cm^−1^ and 1582 cm^−1^ are due to the asymmetric degenerated stretching of CN_3_ and the degenerated scissoring vibration of NH_2_, respectively [36,39]. Particularly, the peaks at 1351 cm^−1^ and 908 cm^−1^ associated with free CH_3_SO_3_H are not observed in [Gdm][OMs], implying no detectable excess acid in the product [40,41]. Moreover, the broadening of the vibrational bands between 3000 cm^−1^ and 3500 cm^−1^ implies a hydrogen bonding network [42,43]. The absorption bands at around 2825, 2755, and 2236 cm^−1^ may be attributed to the N−H or N^+^−H stretching vibration, thus also proving the salt formation [44].

The DSC thermogram of [Gdm][OMs] is shown in Figure 2b. The experimental conditions and results of DSC measurements in comparison with the literature [15,16] for [Gdm][OMs] are summarized in Table 2. [Gdm][OMs] exhibits distinct melting and solidification peaks during the heating and cooling cycles. It shows a very sharp and symmetrical endothermic melting peak with an accurate melting point (*T*_m_) of 207.6 °C and a specific latent heat of fusion of 183.0 J g^−1^ (Figure 2b), indicating the high purity of as-prepared [Gdm][OMs]. The values of *T*_m_ and Δ_fus_*H* agree well with the values reported by MacFarlane et al. (*T*_m_ = 208 ± 1 °C; Δ_fus_*H* = 190 ± 9.5 J g^−1^) [15,16].

### 4.2. Vapor Pressure and Enthalpy of Vaporization

**Figure 3:**
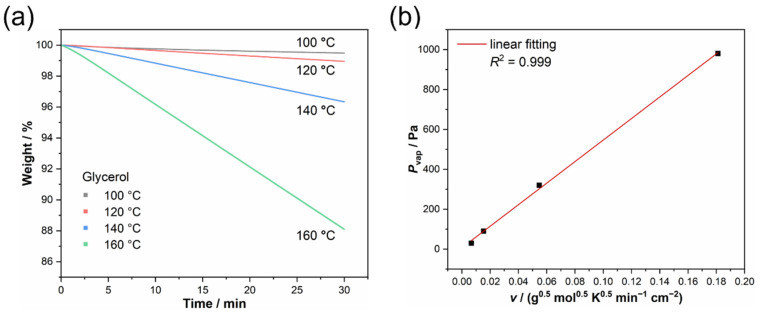
(**a**) IGA traces of glycerol at different temperatures; (**b**) the plot of *P*_vap_ vs. *v* for glycerol.

Glycerol is used as the calibrator for the isothermogravimetric method to determine the constant *k*_vap_ (Equation (2)). Based on the IGA traces of glycerol at different temperatures (Figure 3a), the distinct mass loss rates of glycerol, the corresponding coefficient of determination *R*^2^ (all of them exceeds 0.98) for linear regression, and the values of *v* were obtained and are listed in Table 3. Using the reported vapor pressure data of glycerol [45,46], the curve of *P*_vap_ vs. *v* is plotted in Figure 3b. Obviously, there is a linear relationship between *P*_vap_ and *v,* and the constant *k*_vap_ was evaluated from the slope as 5397 Pa min cm^2^ g^−0.5^ mol^−0.5^ K^−0.5^, with an excellent coefficient of determination (*R*^2^) of 0.999.

**Figure 4:**
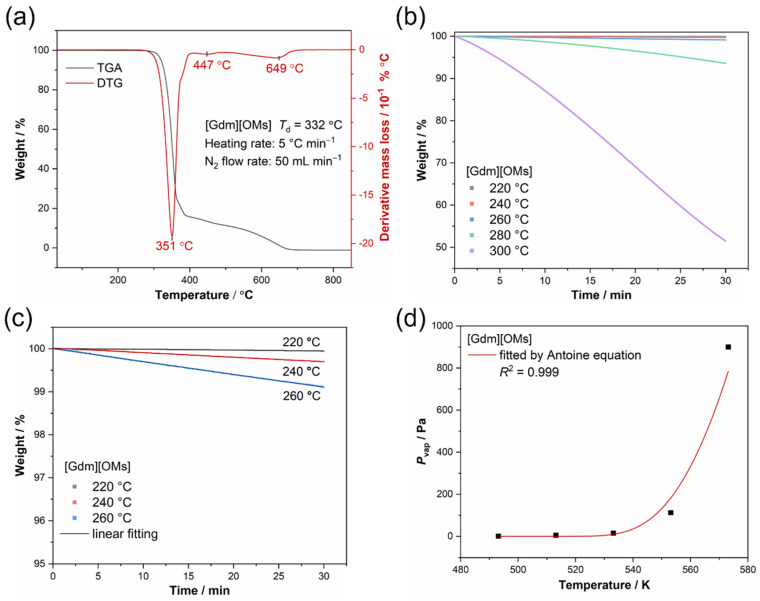
(**a**) TGA trace and DTG curve of [Gdm][OMs]; (**b**) IGA traces of [Gdm][OMs] at different temperatures; (**c**) Selected IGA traces and the linear fitting curves at 220 °C, 240 °C and 260 °C for [Gdm][OMs]; (**d**) The fitting curve using Antoine equation for [Gdm][OMs].

The TGA trace of [Gdm][OMs] at 5 K min^−1^ in nitrogen atmosphere (Figure 4a) indicates that the onset temperature of weight loss of [Gdm][OMs] is as high as 332 °C (605.15 K), revealing its thermal stability over a wide temperature range. It also confirms the ultra-low water content as no loss of water was observed. Furthermore, derivative thermogravimetry (DTG), in which the rate of weight changes of [Gdm][OMs] upon heating is plotted against temperature, demonstrates three thermal events associated with vaporization or decomposition at higher temperatures of 351 °C, 447 °C, and 649 °C successively. Figure 4b shows the IGA traces of [Gdm][OMs] at 220 °C, 240 °C, 260 °C, 280 °C, and 300 °C, respectively. Clearly, the weight loss rate of [Gdm][OMs] increases as the temperature increases, demonstrating that the higher the temperature is, the easier it is for [Gdm][OMs] to evaporate. Table 4 shows the linear fitting results of the IGA traces with *R*^2^ in the range of 0.939–0.999.

The measured vaporization rates and the calculated values of *v* (*v* = [(−d*m*/d*t*)/*S*](*T*/*M*_w_)^0.5^) are listed in Table 4. The vapor pressures of [Gdm][Oms] at 220 °C, 240 °C, 260 °C, 280 °C, and 300 °C are then calculated via Equation (2) (*P*_vap_ = *k*_vap_*v*) thanks to the calibrated value of the constant *k*_vap_ (5397 Pa min cm^2^ g^−0.5^ mol^−0.5^ K^−0.5^) using glycerol. Note that *k*_vap_ is dependent on the apparatus and experimental atmosphere. The derived vapor pressure of [Gdm][OMs] at 533.15 K (260 °C) is 15.23 Pa (Table 4), which is much lower than that of 1-butyl-3-methylimidazolium bis(trifluoromethanesulfonyl)imide ([C_4_mim][NTf_2_]) (*P*_vap_ = 4660 Pa at 517.45 K), indicating that this protic organic salt has very low volatility even at high temperatures [47]. The ultra-low vapor pressure means less loss and better durability of [Gdm][OMs] as a PCM and also implies a simpler design, a lower cost, and higher safety for the containers in terms of pressure vessels.

The three Antoine constants are obtained by fitting *P*_vap_ vs. *T* using the Antoine equation (Figure 4d, *R*^2^ = 0.999), where *A*, *B*, and *C* are 10.99, 344.58, and −493.64, respectively. The temperature dependence of vapor pressures ln *P*_vap_ (Pa) = 10.99 − 344.58/(*T* (K) − 493.64) in the temperature range 493.15–573.15 K is thus acquired. The approximated semi-empirical equation indicates the exponential relationship between the vapor pressure and the temperature of [Gdm][OMs]. Moreover, the vapor pressures of [Gdm][OMs] between 220 °C and 300 °C can thus be interpolated.

**Figure 5:**
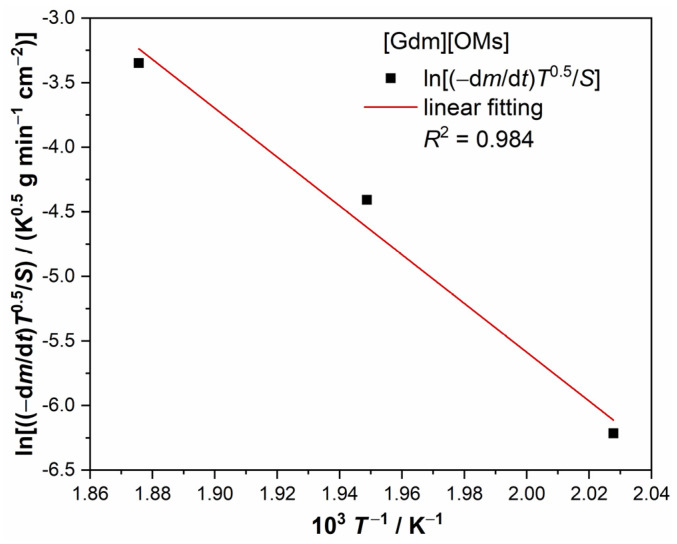
The linear fit based on the linear weight loss rates at 220 °C, 240 °C, and 260 °C, calculated from Figure 4c according to Equation (4). The fitting yields a slope of −Δ_vap_*H*/(1000*R*), where Δ_vap_*H* is the enthalpy of vaporization and *R* is the gas constant.

Figure 5 shows a linear fit based on the linear weight loss rates at 220 °C, 240 °C, and 260 °C calculated from Figure 4c (also shown in Table 4) according to Equation (4). The calculated Δ_vap_*H* at the average temperature of 513.15 K (240 °C) for [Gdm][OMs] is 157.10 ± 20.10 kJ mol^−1^. The estimated value of Δ_vap_*H* is higher than those of [C_4_mim][NTf_2_] (Δ_vap_*H* = 118.5 ± 0.4 kJ mol^−1^ at 496 K) and guanidinium nonaflate ([Gdm][NfO], Δ_vap_*H* = 120.9 kJ mol^−1^ at 523 K), which again proves that [Gdm][OMs] has essentially low volatility [27,31].

**Figure 6:**
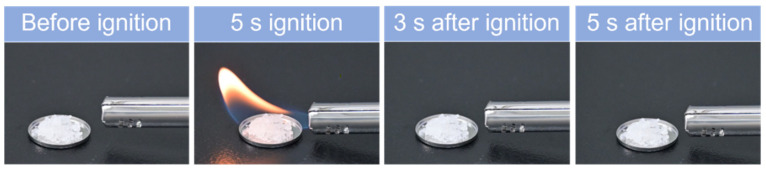
Photographs of the ignition tests of [Gdm][OMs].

Finally, the flammability test (Figure 6) indicates that [Gdm][OMs] cannot be ignited after 5 s of combustion in air and will not explode when exposed to open flames, either. This demonstrates that [Gdm][OMs] is non-flammable and has excellent thermal safety.

## 5. Conclusions

Pure guanidinium methanesulfonate ([Gdm][OMs]) was prepared by a slight deviation from the stoichiometric ratio of methanesulfonic acid to guanidinium carbonate (i.e., the acid is slightly excess), followed by heating, stirring, vacuum drying, and then recrystallization. The elemental analysis, infrared spectrum measurement, thermogravimetric analysis (TGA), and differential scanning calorimetry (DSC) all confirmed its high purity. TGA confirms its thermal stability over a wide temperature range while it also indicates three thermal events due to vaporization or decomposition between 350 °C and 650 °C. Using isothermogravimetric analysis, the Antoine equation was successfully employed to describe its vapor pressure as a function of temperature between 493.15 K and 573.15 K: ln *P*_vap_ (Pa) = 10.99 – 344.58/(*T* (K) – 493.64), indicating the vapor pressures ranging from 0.87 Pa at 220 °C to 899.98 Pa at 300 °C. Furthermore, its enthalpy of vaporization is derived as Δ_vap_*H* = 157.10 ± 20.10 kJ mol^−1^ at the average temperature of 240 °C via the Clausius–Clapeyron equation. In summary, [Gdm][OMs] exhibits a series of additional advantages, including essentially low volatility, high enthalpy of vaporization, and non-flammability. This work is expected to offer data support, methodologies, and insights for the application of organic salts as a new type of phase change materials in thermal energy storage and other industrial fields.

## Figures and Tables

**Table 1:** Some PCMs with melting points in the intermediate temperature range (100–230 °C).

PCMs	*T*_m_ (°C)	Δ_fus_*H* (J g^−1^)	Δ*T* (°C) ^[b]^
MgCl_2_·6H_2_O [6]	123	117	41
Indium [7]	157	29	/
Selenium [7]	220	86	/
Erythritol [8,9]	118	340	86
D-dulcitol [11]	186	334	/
C_3_(mim)_2_(Br)_2_ [12]	173	116	46
[Pzy][OMs] [13]	168	160	/
[Pzy][OTf] [13]	147	17 ^ss^, 24 ^ss^, 27	/
[Pzy][C_6_H_5_SO_3_] [13]	137	105	/
[PyH][OMs] [14]	180	21 ^ss^, 77	16
[PyH][OTf] [14]	226	24 ^ss^, 38	41
[pyH][C_6_H_5_SO_3_] [14]	135	123	80
[Gdm][OMs] [15,16]	208	190	/
[Gdm][OTf] [15,16]	160	6 ^ss [a]^, 130	/
[Gdm][C_6_H_5_SO_3_] [15,16]	210	138	/
C_2_(mim)_2_(Br)_2_ [17]	188	116	24
C_2_(mim)_2_(PF_6_)_2_ [17]	191	109	/

^[a]^ ss: solid–solid phase transition. ^[b]^ Δ*T*: degree of supercooling, i.e., difference between *T*_m_ and crystallization temperature.

**Table 2:** Experimental conditions and results of DSC measurements for [Gdm][OMs].

Experimental Conditions and Results	This Work	Literature [15,16]
Sample mass	8.34 mg	3~8 mg
Scanning rate	5 °C min^−1^	10 °C min^−1^
Atmosphere and flow rate	N_2_, 60 mL min^−1^	N_2_ (flow rate not mentioned)
Measurement temperature range	25~240 °C	−40~220 °C
DSC instrument	STARe DSC 3, Mettler-Toledo (with 120 thermocouples)	DSC TA Q200 calorimeter (TA Instruments)
Sample condition	powders	NOT MENTIONED
Crucible	40 μL aluminum pan (sealed)	NOT MENTIONED
Data selection	the second run of the DSC cycle	the second run of the DSC cycle
*T*_m_ (peak maximum)	207.6 °C	208 ± 1 °C
Δ_fus_*H*	183.0 J g^−1^	190 ± 9.5 J g^−1^

**Table 3:** Vaporization rates, the values of *v*, and the reported vapor pressures of glycerol.

*T*/°C	*T*/K	(−d*m*/d*t*)/*S*/g min^−1^ cm^−2^	*R* ^2^	*v*/g^0.5^ mol^0.5^ K^0.5^ min^−1^ cm^−2^	*P*_vap_/Pa [45,46]
100	373.15	3.32 × 10^−3^	0.980	6.69 × 10^−3^	30.0
120	393.15	7.42 × 10^−3^	0.999	1.53 × 10^−2^	90.0
140	413.15	2.58 × 10^−2^	0.999	5.47 × 10^−2^	320
160	433.15	8.36 × 10^−2^	0.999	1.81 × 10^−1^	980

**Table 4:** Vaporization rates, the values of *v,* and derived vapor pressures of [Gdm][OMs].

*T*/°C	*T*/K	(−d*m*/d*t*)/*S*/g min^−1^ cm^−2^	*R* ^2^	*v*/ g^0.5^ mol^0.5^ K^0.5^ min^−1^ cm^−2^	*P*_vap_/Pa
220	493.15	9.01 × 10^−5^	0.939	1.61 × 10^−4^	0.87
240	513.15	5.39 × 10^−4^	0.998	9.79 × 10^−4^	5.29
260	533.15	1.52 × 10^−3^	0.999	2.82 × 10^−3^	15.23
280	553.15	1.10 × 10^−3^	0.980	2.08 × 10^−2^	112.26
300	573.15	8.68 × 10^−2^	0.996	1.67 × 10^−1^	899.98

## Data Availability

The raw data supporting the conclusions of this article will be made available by the authors on request.
